# Redundant functions of the SLC5A transporters Rumpel, Bumpel, and Kumpel in ensheathing glial cells

**DOI:** 10.1242/bio.059128

**Published:** 2022-01-18

**Authors:** Kerem Yildirim, Bente Winkler, Nicole Pogodalla, Steffi Mackensen, Marie Baldenius, Luis Garcia, Elke Naffin, Silke Rodrigues, Christian Klämbt

**Affiliations:** 1 Institute for Neuro- and Behavioral Biology, University of Münster, Badestr. 9, 48149 Münster, Germany; 2 Centre for Organismal Studies (COS) Heidelberg, University of Heidelberg, Im Neuenheimer Feld 230, 9120 Heidelberg, Germany

**Keywords:** *Drosophila*, Ensheathing glia, SLC5A transporters, Redundancy, *CG9657*, *CG6723*, *CG42235*

## Abstract

Neuronal processing is energy demanding and relies on sugar metabolism. To nurture the *Drosophila* nervous system, the blood-brain barrier forming glial cells take up trehalose from the hemolymph and then distribute the metabolic products further to all neurons. This function is provided by glucose and lactate transporters of the solute carrier (SLC) 5A family. Here we identified three SLC5A genes that are specifically expressed in overlapping sets of CNS glial cells, *rumpel*, *bumpel* and *kumpel*. We generated mutants in all genes and all mutants are viable and fertile, lacking discernible phenotypes. Loss of *rumpel* causes subtle locomotor phenotypes and flies display increased daytime sleep. In addition, in *bumpel kumpel* double mutants, and to an even greater extent in *rumpel bumpel kumpel* triple mutants, oogenesis is disrupted at the onset of the vitollegenic phase. This indicates a partially redundant function between these genes. Rescue experiments exploring this effect indicate that oogenesis can be affected by CNS glial cells. Moreover, expression of heterologous mammalian SLC5A transporters, with known transport properties, suggest that Bumpel and/or Kumpel transport glucose or lactate. Overall, our results imply a redundancy in SLC5A nutrient sensing functions in *Drosophila* glial cells, affecting ovarian development and behavior.

## Abstract (precis)

**Summary:** The *Drosophila* genes rumpel, bumpel and kumpel, that encode SLC5A proteins expressed in overlapping sets of CNS glial cells act in partially redundant manner during locomotor control and oogenesis.

## INTRODUCTION

Cells require a constant energy supply to function. Metabolic activity is in particular high in the nervous system, where large amounts of ATP is needed to maintain synaptic transmission and cope with the resulting changes in membrane potential. This is reflected by energy consumption as the mammalian brain accounts for 20% of the total resting oxygen consumption although comprising only 2% of the body's weight (Karbowski, 2007; Mink et al., 1981; Nortley and Attwell, 2017). This energy demand is even greater in young brains and is similarly found in the invertebrate nervous system (Harris et al., 2012; Laughlin et al., 1998; Mink et al., 1981; Tsacopoulos et al., 1988).

In the vertebrate nervous system, glucose is the predominant metabolite supplying the brain with energy. Glucose circulates in the blood stream and is delivered to the different organs. The brain is metabolically separated from circulation by the blood-brain barrier, which is comprised of endothelial cells that form occluding tight junctions (Abbott et al., 2006; Tam and Watts, 2010; Tietz and Engelhardt, 2015; Zlokovic, 2008). Endothelial cells take up glucose from the blood stream via the Glut1 transporter. From the endothelial cells, glucose is then shuttled to astrocytes and neurons by different glucose transporters. While endothelial cells and astrocytes express differentially glycosylated forms of the glucose transporter Glut1, neurons predominantly express Glut3 (Barros et al., 2007; Vannucci et al., 1997). To match fluctuating neuronal energy demands, glial cells are able to sense synaptic activity. The Astrocyte Neuron Lactate Shuttle (ANLS) hypothesis, initially established for the mammalian brain provides an elegant model explaining how the flux of small C3 metabolites is regulated in the brain (Magistretti and Allaman, 2018; Pellerin and Magistretti, 1994; Pellerin et al., 2007).

In contrast to vertebrates, invertebrates do not have a vascular system. Instead, the hemolymph, the blood equivalent tissue of invertebrates, is found in all body cavities and immerses the entire nervous system. The predominant sugar in hemolymph is trehalose, a non-reducing disaccharide composed of two glucose molecules linked in an α,α-1,1-glycosidic manner, acting as the prime energy source (Wyatt and Kalf, 1957). As in vertebrates, the nervous system is metabolically separated from the remaining body by the blood-brain barrier (Carlson et al., 2000; Limmer et al., 2014; Mayer et al., 2009). In *Drosophila* the blood-brain barrier is established by perineurial and subperineurial glial cells (Stork et al., 2008). Perineurial cells express trehalose transporters and participate in maintaining the energy homeostasis of the brain (McMullen et al., 2020; Volkenhoff et al., 2015). The subperineurial glial cells block paracellular diffusion by interdigitating cell–cell processes and the formation of septate junctions (Babatz et al., 2018; Bundgaard and Abbott, 2008; Schwabe et al., 2005; Stork et al., 2008). Trehalose is taken up from the circulation by the Tret1-1 transporter, which is expressed by perineurial glial cells. In addition, MFS3 and Pippin are involved in carbohydrate transport in the perineurial glia. Interestingly, MFS3 or Pippin null mutants are rescued via compensatory upregulation of Tret1-1, another blood-brain barrier carbohydrate transporter, while RNAi-mediated knockdown of *Mfs3* and *pippin* is not compensated for (McMullen et al., 2020). Trehalose is subsequently metabolically processed through glycolysis. Lactate and alanine are then delivered to neurons by as yet poorly characterized transport mechanisms (Delgado et al., 2018; González Gutiérrez et al., 2019; Volkenhoff et al., 2015).

In general metabolite transport is mediated by members of the solute carrier protein (SLC) family, which allow either facilitated, or active transport into the cell. The SLC superfamily constitutes approximately 400 genes grouped into more than 50 families and many of its members are expressed in the brain (Bai et al., 2017; Morris et al., 2017). Two of these transporter families have been identified to be involved in glucose transport. The solute carrier proteins of the SLC2A family (Glut1-14) mediate facilitated glucose diffusion across the plasma membrane, whereas members of the SLC5A family (SGLT1-5) can transport glucose, fructose, lactate or pyruvate in a sodium gradient-dependent manner (Mueckler and Thorens, 2013; Wright, 2013; Wright et al., 2011).

In *Drosophila*, it is long known from deoxyglucose labeling experiments that glucose can be taken up by neurons in the brain (Buchner et al., 1979). Moreover, recent experiments using a FRET-based glucose sensor expressed in neurons of the *Drosophila* central nervous system (CNS) demonstrated that neurons are able to take up glucose in the same manner as glial cells (Volkenhoff et al., 2018). The fly orthologue of the mammalian Glut1 transporter is expressed exclusively in neurons and is not expressed in the blood-brain barrier (Volkenhoff et al., 2018).

The cellular route and transporters involved in delivery of trehalose, as well as glycolytic derived products to neurons remains elusive. The *Drosophila* nervous system comprises a relatively small set of well-defined glial cells, which establish a glial network that connects glial cells of the blood-brain barrier with the synaptic neuropil (Freeman, 2015; Yildirim et al., 2018). The cortex glial cells engulf neuronal cell bodies (Coutinho-Budd et al., 2017; Spéder and Brand, 2018). Axons and dendrites are located in the neuropil, which is infiltrated by numerous fine cell processes of the astrocyte-like glial cells (MacNamee et al., 2016; Peco et al., 2016; Stork et al., 2014). These cells modulate synaptic activity by participating in neurotransmitter homeostasis and the secretion of additional modulatory factors (Liu et al., 2014; Ma et al., 2016; Sengupta et al., 2019). The cell bodies of the astrocyte-like glia cells are found at the boundary of the neuropil, next to the ensheathing glial cell bodies (Peco et al., 2016). Ensheathing glia encase the entire neuropil and also participate in the modulation of locomotor activity, as well as in the regulation of sleep (Otto et al., 2018; Stahl et al., 2018).

Assuming that trehalose is taken up from the hemolymph at the blood-brain barrier, we hypothesize that further transport of its metabolic products (glucose, pyruvate or lactate) within the brain must be coordinated by other still elusive transporters. Here we uncover such transporters. We report the identification of three SLC5A family members [*rumpel*, *bumpel* (for *brother of rumpel*) and *kumpel* (for *kin of rumpel*)] that are specifically expressed by inner CNS glial cells and act in highly redundant manner to support neuronal function. Loss-of-function mutants of *rumpel*, *bumpel* or *kumpel* cause only very subtle behavioral phenotypes, whereas double and triple mutants showed behavioral phenotypes as well as female sterility demonstrating redundant gene functions.

## RESULTS

### Identification of predicted sugar transporters expressed in the fly brain

**Fig. 1.**
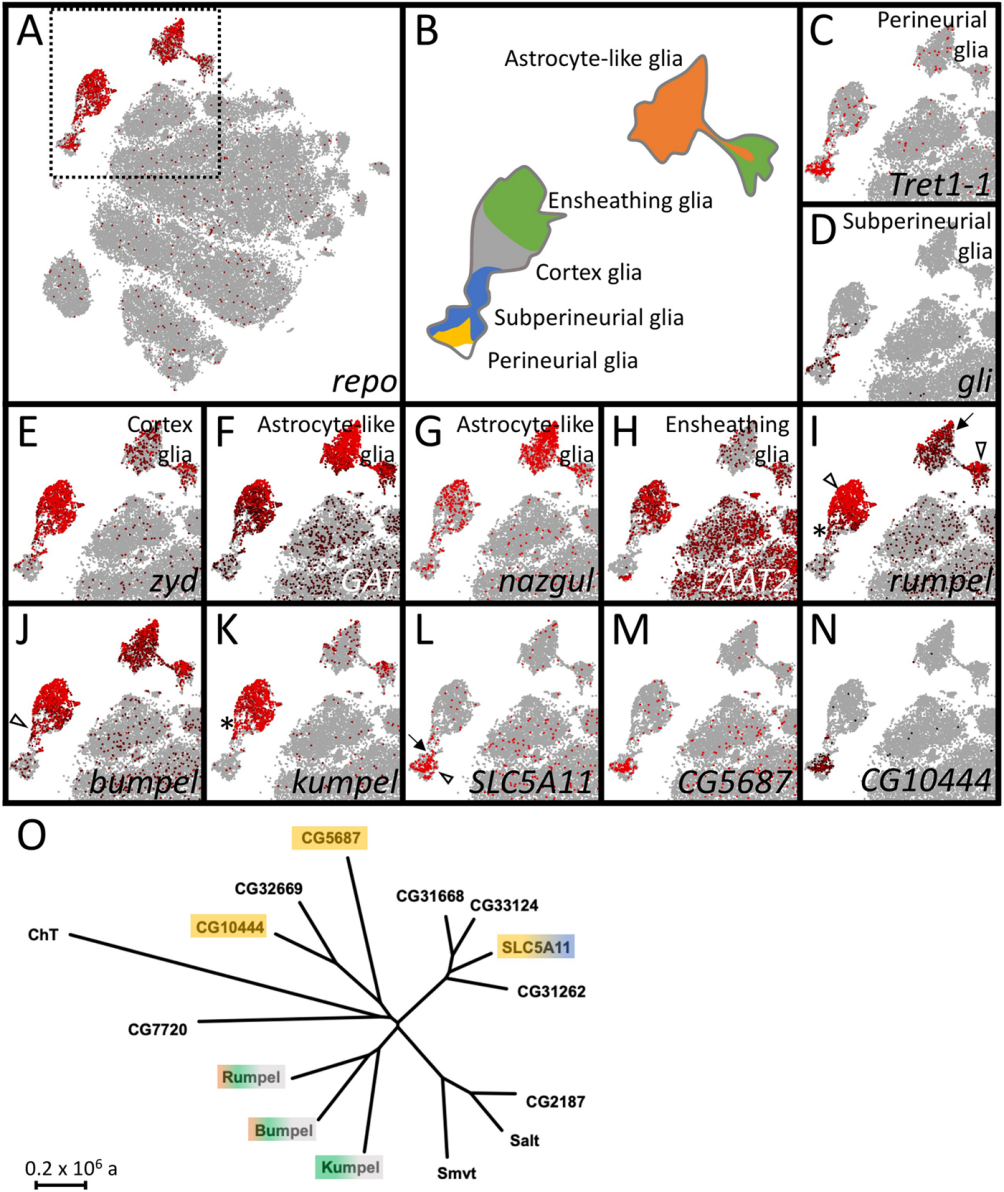
**The expression of SLC5 family members in the adult brain.** (A–N) Single cell RNA sequencing data in the SCENIC representations of the 57 K scRNA seq data set (Davie et al., 2018). SCope analysis for the genes indicated in each bottom right corner is shown. Each dot represents a single cell. The color coding indicates the expression level. Red: strong expression, black: low expression. Grey: no expression. (A,B) *repo* expression marks glial cell clusters that can be assigned as perineurial (yellow), subperineurial (blue), cortex (grey), ensheathing (green) or astrocyte-like glial cells (orange) according to marker gene expression as shown in (C–H). (I–N) Expression of SLC5 family members that show expression in *Drosophila* glia. (O) Dendrogram of the evolutionary relationships of the different SLC5 family members of *Drosophila*. The color shading indicates expression in the respective glial cell type (see B). The scale bar represents 2×10^5^ years of evolutionary distance.

Energy homeostasis in the brain is mediated by carbohydrate provision. Sugars are taken up from the hemolymph at the blood-brain barrier forming glial cells and then must be shuttled to neurons by other glially expressed transporters. The *Drosophila* genome encodes 15 predicted glucose and monocarboxylate transporter proteins of the SLC5A family, which are strong candidates to organize sugar distribution in the nervous system (Featherstone, 2011) (Fig. 1). We thus searched for SLC5A members that are expressed by glial cells inside the CNS.

Using recent single cell RNA sequencing data (Davie et al., 2018) expression of all predicted SLC5A sugar transporters can be traced to specific glial cell types (Fig. 1A–I). Expression of the glial cell marker Repo defines all glial cells in the adult fly brain (Halter et al., 1995). The different glial subtypes are characterized by expression of specific genes [perineurial glial cells: *tret1-1* (Volkenhoff et al., 2015), subperineurial glial cells: *gliotactin* (Auld et al., 1995; Babatz et al., 2018), cortex glia: *zydeco* (Melom and Littleton, 2013), astrocyte-like glial cells: *GAT* and *nazgul* (Ryglewski et al., 2017; Stork et al., 2014) and ensheathing glial cells: *EAAT2* (Peco et al., 2016)]. The different glial subtypes cluster in distinct groups of cells (Davie et al., 2018) (Fig. 1A–I).

In the adult brain, *CG9657* is expressed most strongly in the ensheathing glia cluster but in addition some cortex glia and astrocyte-like glial cells express *CG9657* (Fig. 1A–I). *CG9657* was also identified in an RNAi-based screen for adult locomotor deficits using a construct without any predicted off-target (Dietzl et al., 2007; Schmidt et al., 2012). Due to an adult paralysis phenotype the gene was named *rumpel*, in honor of the slow-moving character of the Sesame Street.

In addition, *CG6723* and *CG42235* encode highly related proteins that are expressed in very similar set of glial cells in the adult CNS. We thus named *CG6723* as *brother of rumpel* (*bumpel*) and the gene *CG42235* as *kin of rumpel* (*kumpel*).

### *rumpel* is expressed by ensheathing and astrocyte-like glia

**Fig. 2.**
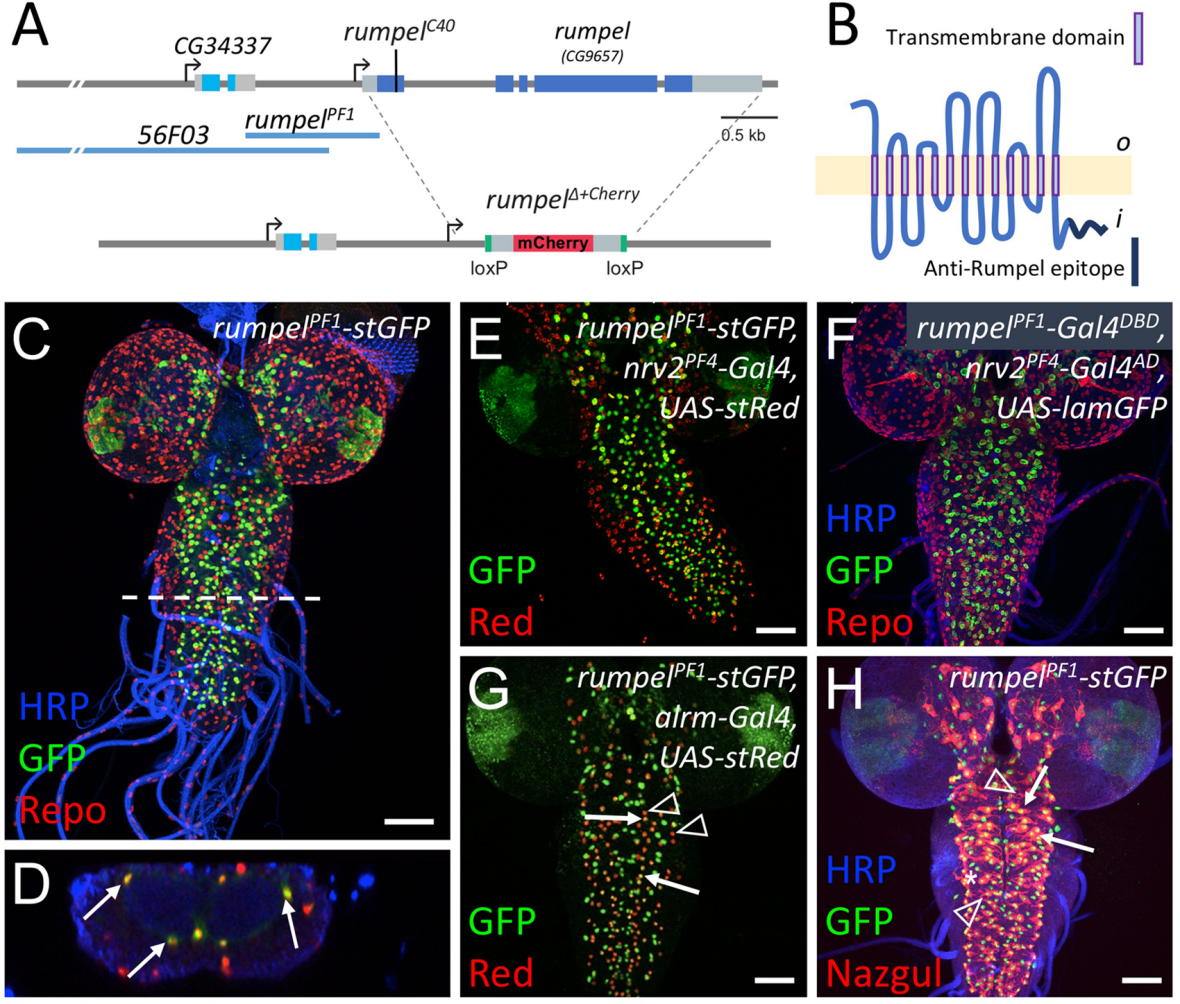
***rumpel*-PF1 induces an expression in the neuropil-associated glial cells.** (A) Schematic representation of the *rumpel* (*CG9657*) locus on the X-chromosome. Exons are shown in boxes, *rumpel* coding exons are in dark blue, *56F03* and *rumpel PF1* denote enhancer elements that direct expression in ensheathing glia. The position of the CRISPR-induced premature stop codon in amorphic allele (*rumpel^C40^*) and the *rumpel* locus replacement with *attP-loxP-Cherry-loxP* in (*rumpel*^Δ*+Cherry*^) is indicated. (B) The Rumpel protein is predicted to have 13 membrane (light yellow) spanning domains. The peptide sequence used to immunize rabbits is highlighted in dark blue. o, outside; i, inside. (C–E,G,H) Specimens are stained for promoter fragment induced expression of StingerGFP (stGFP, green). (E,G) RedStinger (stRed, red). (F) LaminGFP (lamGFP, green). (C,D) Glial nuclei are stained for Repo protein localization (red). (H) Astrocyte-like glial cells are stained for Nazgul protein localization (red). Neuronal membranes are shown in blue (HRP staining). (C) *rumpel* promoter fragment PF1 (*rumpel^PF1^*) induces stGFP expression in Repo positive cells in the third instar larval brain. White dashed line indicates the position of the orthogonal section shown in D. (D) Glial cells in the position of ensheathing glia are indicated by arrows. No expression is observed in surface associated glial cells. (E) *rumpel^PF1^* induced stGFP expression overlaps with the *nrv2* induced RedStinger expression. (F) Split Gal4 directed expression of LamGFP is found in ensheathing glial cells [*rumpel^PF1^-Gal4DBD, nrv2PF4-Gal4^AD^, UAS-lamGFP*]. (G) *rumpel^PF1^* induced stGFP expression is found in some astrocyte-like glial cells labelled by *alrm* induced stRed expression (compare arrows with arrowheads). (H) *rumpel^PF1^* induced stGFP expression in Nazgul positive astrocyte-like glial cells (arrows). The asterisk denotes ensheathing glial nuclei, the arrowhead denotes astrocytes not activating the *rumpel^PF1^* enhancer. Scale bars: 50 µm.

The *rumpel* gene is situated on the X-chromosome and encodes a predicted sugar transporter protein of the sodium solute symporter 5A (SLC5A) family with 13 transmembrane domains (Fig. 2A,B; Fig. S1). To further identify the cells expressing *rumpel* we dissected the *rumpel* promotor region. A 1.1 kb long enhancer fragment designated as *rumpel^PF1^* (Fig. 2A) directs specific expression in the nervous system only in cells that are Repo positive (Fig. 2C,D). Based on their location around the neuropil, the *rumpel* expressing cells may correspond to ensheathing glial cells and/or astrocyte-like glial cells. To further test which cell type activates the *rumpel* enhancer we crossed the *rumpel^PF1^-stGFP* construct into a genetic background directing the expression of a red nuclear marker in the ensheathing and cortex glial cells (*rumpel^PF1^-stGFP, nrv2-Gal4; UAS-stRed*) (Fig. 2E). Most *rumpel* expressing neuropil-associated cells also show *nrv2-Gal4* activity, suggesting that *rumpel* positive cells are expressed in ensheathing glial cells. This notion is corroborated by split-Gal4 experiments where we co-expressed the Gal4-DNA-binding domain in the *rumpel* pattern and the Gal4 activation domain in the *nrv2* pattern (*rumpel^PF1^-Gal4^DBD^, nrv2^PF4^-Gal4^AD^*) (Fig. 2F). To test whether Rumpel is also expressed by astrocyte-like glial cells, we analyzed animals expressing GFP under the control of the *rumpel* enhancer and dsRed under the control of the *alrm* enhancer, which is active in astrocytes. In the larval central nervous system of such animals, we noted frequent coexpression (Fig. 2G), suggesting that Rumpel is also expressed by astrocyte-like glial cells. Similarly, when we stained *rumpel^PF1^-stGFP* larval brains with anti-Nazgul antibodies we noted a partial overlap (Fig. 2H). The *rumpel^PF1^* fragment overlaps with the enhancer fragment *56F03* generated by the FlyLight project (Jenett et al., 2012; Li et al., 2014), which is reported to direct expression in ensheathing glia (Li et al., 2014; Otto et al., 2018; Peco et al., 2016) (Fig. 2A). This indicates that the critical enhancer elements are located in the 700 bp overlap of the two enhancer fragments.

### Rumpel protein expression in the nervous system

**Fig. 3.**
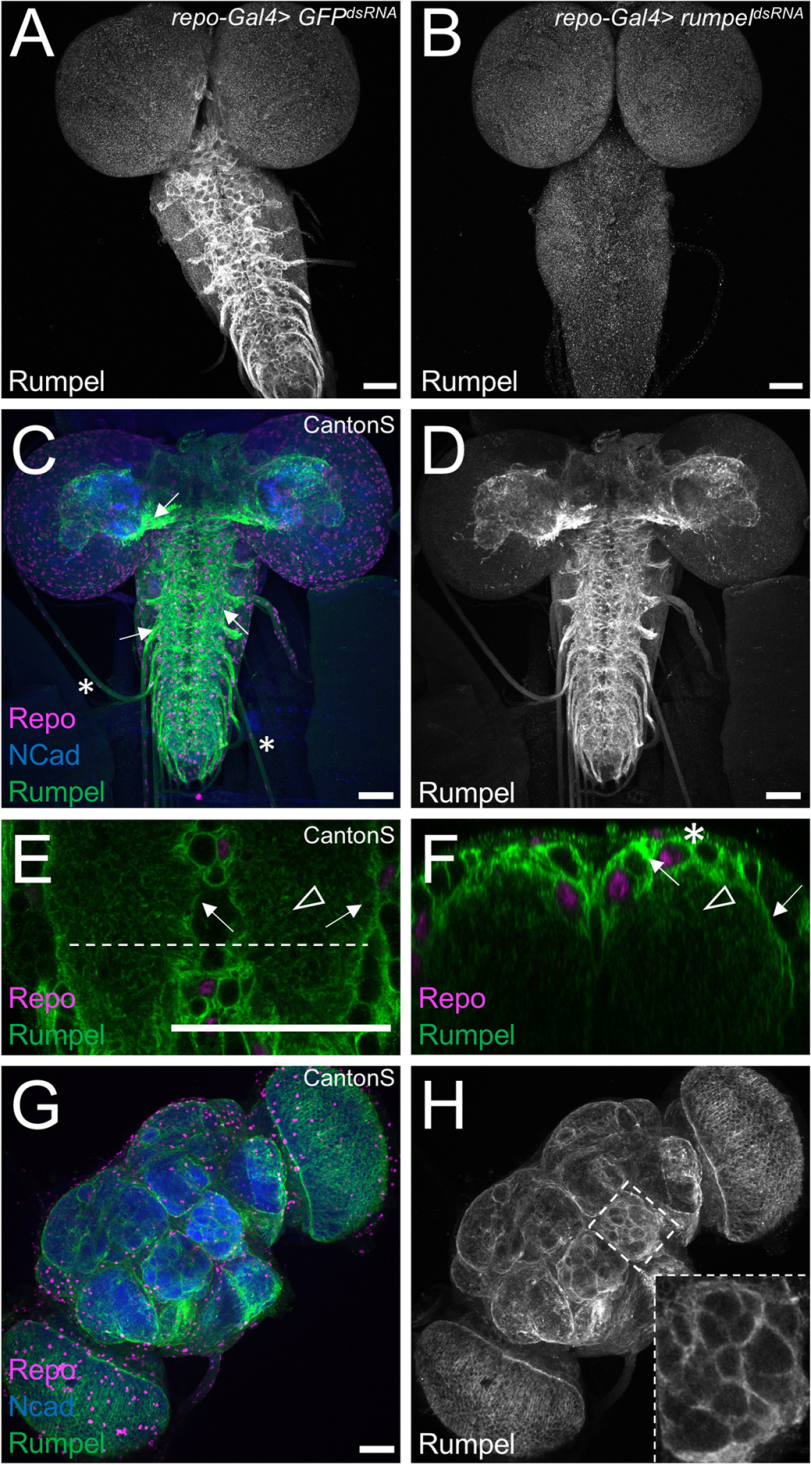
**Rumpel protein is expressed in the neuropil-associated glial cells.** All specimens are stained for Repo localization to define glial nuclei (magenta), for N-Cadherin localization to visualize axonal and dendritic cell membranes (blue) and for Rumpel protein localization (green/grey). (A–F) Third instar larval brains and (G,H) adult brain. (A) In control animals [*repo-Gal4, UAS-GFP^dsRNA^*] Rumpel protein localizes around the neuropil. (B) Upon expression of *rumpel^dsRNA^* in the all-glial cells [*repo-Gal4, UAS- rumpel^dsRNAv43922^*] no Rumpel protein can be detected, demonstrating the specificity of the anti-Rumpel antibody. (C,D) Rumpel localization is observed surrounding the neuropil (arrows) in a position of the ensheathing glial cells. Very little Rumpel protein is found along larval nerves (asterisks). (E) Image of a single confocal plane through a third instar larval ventral nerve cord. Rumpel localizes to ensheathing glial cell membrane (arrow) and to cell processes of astrocyte-like glial cells (arrowhead). The dashed line indicates the position of the orthogonal section shown in F. (F) Rumpel localizes to ensheathing glial cells (arrows) and astrocytic processes in the neuropil (arrowhead). Note, the pronounced cortex-glial cell like ramifications of the ensheathing glia dorsally to the neuropil (asterisk). (G,H) Rumpel localizes around the neuropil in adult brains at a position of the ensheathing glia (inset: antennal lobe). Scale bars: 50 µm.

To determine the localization of the Rumpel protein, we generated an anti-peptide antiserum directed against the C-terminal most amino acids (Fig. 2B). The specificity of the antiserum is demonstrated following pan-glial silencing of *rumpel* expression using RNAi (Fig. 3A,B). In the third instar larvae, no expression is discernible outside the CNS, which matches RNAseq expression data (Brown et al., 2014; Graveley et al., 2011). Within the nervous system, Rumpel localizes to cell membranes of neuropil associated cells in the developing brain lobes as well as in the ventral nerve cord (Fig. 3A–F, arrows). In addition, some Rumpel protein can be found within the neuropil (Fig. 3E,F, arrowheads). Very low levels of Rumpel protein are detected along the peripheral abdominal nerves that connect the CNS with the periphery. In adults, Rumpel expression is also found prominently in the ensheathing glial cells (Fig. 3G,H).

To further characterize the Rumpel expressing glial cells, we performed glial cell type specific silencing experiments. Following suppression of *rumpel* using *nrv2-Gal4*, which strongly suppresses in ensheathing glial cells and less so in cortex and astrocyte-like glial cells, we noted a complete lack of Rumpel protein localization (Fig. S2A,D). Following suppression in ensheathing glial cell using *83E12-Gal4*, weak Rumpel expression can be detected in the cortex and neuropil, reflecting processes of the astrocyte-like glial cells (Fig. S2B,E). Following suppression of *rumpel* expression, mostly in astrocyte-like glial cells, using *alrm-Gal4*, Rumpel protein can still be detected in the ensheathing glia (Fig. S2C,F).

In conclusion, throughout development of the central nervous system of *Drosophila*, the SLC5A member Rumpel is expressed specifically in glial cells and is most prominently found in ensheathing glial cells with some expression in cortex and astrocyte-like glial cells.

### Generation of *rumpel* mutants

The analysis of Rumpel protein localization demonstrates a specific expression in neuropil associated cells. Moreover, previous RNAi data had suggested a role of *rumpel* in adult locomotor control (Ng et al., 2016; Schmidt et al., 2012). To determine the function of *rumpel*, we generated a loss-of-function allele using the CRISPR/Cas9 methodology, targeting either the first or the fourth exon. The allele *rumpel^C40^* carries a 11 bp deletion leading to a frameshift and subsequent stop of translation after 52 additional amino acids (Fig. 2A). In addition, we replaced the entire *rumpel* gene with an *mCherry* coding sequence using homologous recombination to generate the *rumpel*^Δ*+cherry*^ mutant (see Materials and Methods for a description of all mutants) (Fig. 2A). Both *rumpel* null alleles generated lack detectable expression of the Rumpel protein. Moreover, both *rumpel* null alleles are homozygous viable and show no discernible morphological phenotypes. Likewise, a floxed allele, *rumpel^del^*, shows no detectable phenotypic abnormalities.

### Behavioral analysis of *rumpel* null mutants

**Fig. 4.**
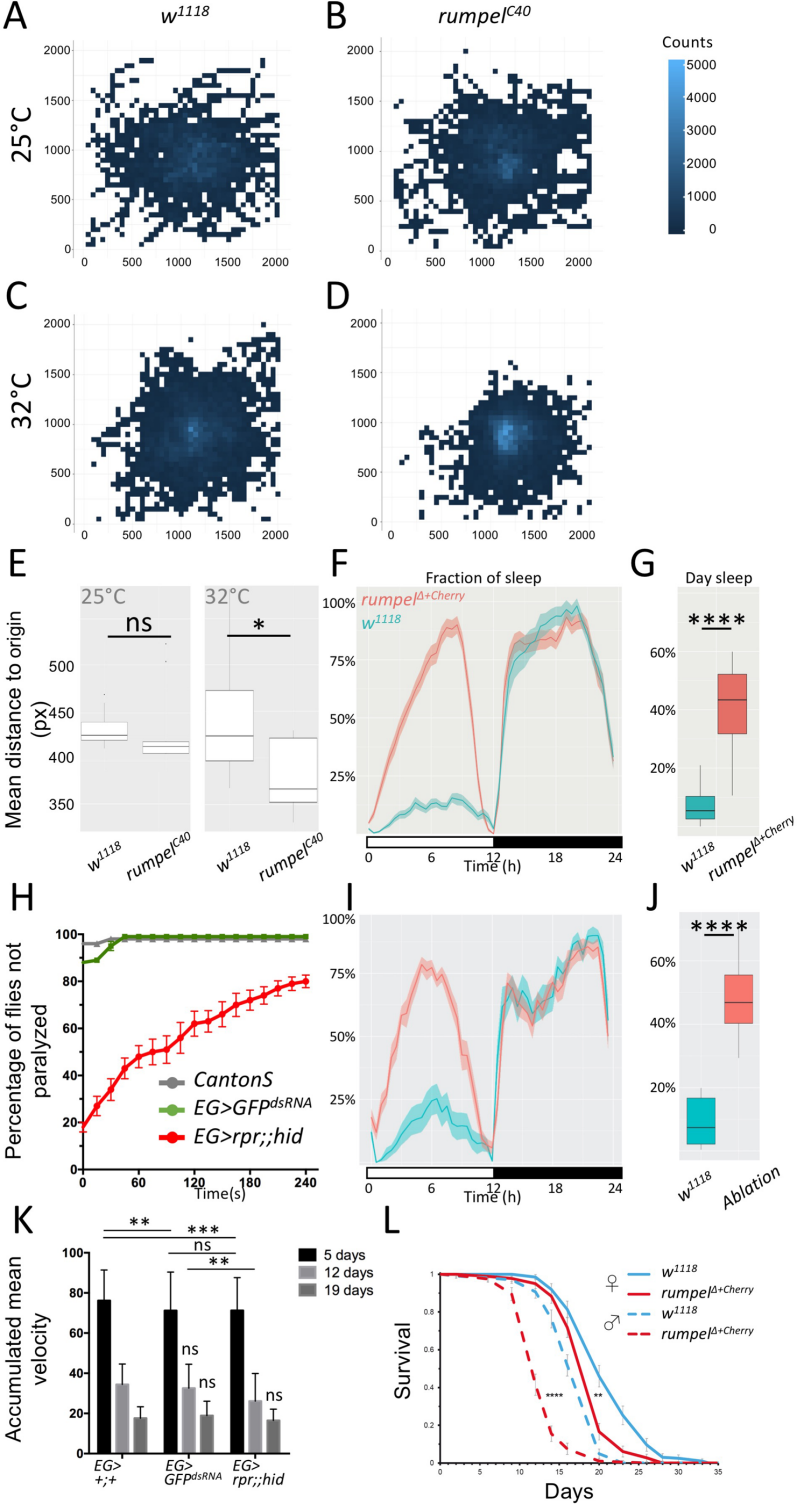
**Behavioral analysis of *rumpel.*** (A–E) 150 third instar larvae of the respective genotypes were recorded in groups of 15 animals for 3 min at 25°C or 32°C, as indicated. Larvae were always placed at the middle of the tracking plate. (A–D) For heatmap analyses, the 2048×2048 px image of the agar plate is divided in 50×50 px squares. The number of larval appearances per square is determined and indicated in blue shading using R. Darker blue colors indicate less frequent appearance, while lighter blue ones more. (A) Heatmap analysis of control *w^1118^* larvae at 25°C. Wild-type larvae crawl in every direction and spread evenly on the agar plate at 25°C (indicated by fewer lighter blue squares). (B) Heatmap analysis of *rumpel^C40^* larvae. *rumpel^C40^* larvae shows wild type-like distribution on the agar plate at 25°C (indicated by similar number of lighter blue squares). (C) Wild-type larvae spread evenly on the agar plate at 32°C. (D) At 32°C *rumpel^C40^* larvae spread less on the agar plate (indicated by more light blue squares in the middle). (E) Quantification of the mean distance to origin of wild-type versus *rumpel^C40^* larvae at 25°C and 32°C. At 25°C no significant difference is indicated (Mann–Whitney *U*-test *P*>0.05, *n*=150). Mean distance to origins of wild type and *rumpel^C40^* are 439.4 and 381.4 px, respectively at 32°C. Wild-type larvae spread significantly more on the agar plate at 32°C compared to *rumpel^C40^* larvae (Mann–Whitney *U*-test *P*=0.023, *n*=150). (F) To monitor the effects of rumpel on sleep behavior *rumpel*^Δ*+cherry*^ flies were backcrossed 10 times to *white^1118^*. The activity of 40 flies was tracked over 7 days in the ethoscope (Geissmann et al., 2017). (G) *rumpel*^Δ*+cherry*^ flies sleep significantly more during the day (*P*=2e-16, Wilcoxon rank sum), whereas night sleep is not affected. (H) Heat shock assay of 5-day-old male and mated female flies of wild type [*Canton S*], [*GMR83E12-Gal4^AD^*; *repo-Gal4^DBD^*, *UAS-GFP^dsRNA^*], [*UAS-rpr*; *GMR83E12-Gal4^AD^*; *repo-Gal4^DBD^*, *UAS-hid*] (each genotype *n*=100). Flies are heat shocked in a water bath for 2 min at 40°C and were immediately recorded at room temperature. Not moving flies lying on their back are considered as paralyzed. Recording was stopped after 240 s. Error bars indicate standard deviation. (I) Average sleep time over seven days summarized for 24 h (shown in %). Flies lacking ensheathing glia show an increased day time sleep compared to the control. (J) Summary of the fraction of time sleeping over 7 days (shown in %). Loss of ensheathing glia leads to an increased sleeping time during the day (*P*=9.991e-07, Wilcoxon rank sum). (K) The rapid iterative negative geotaxis (Ring) assay (Gargano et al., 2005) shows the climbing ability of females with the genotypes: [*GMR83E12-Gal4^AD^*; *repo-Gal4^DBD^*], or [*GMR83E12-Gal4^AD^*; *repo-Gal4^DBD^*, *UAS-GFP^dsRNA^*], or [*UAS-rpr; GMR83E12-Gal4^AD^*; *repo-Gal4^DBD^, UAS-hid*]. The age of tested flies is indicated. Flies are heat shocked in a water bath for 2 min at 40°C and immediately recorded at room temperature for 240 s. Non-moving flies lying on their back are considered to be paralyzed. Both control and ensheathing glia ablated flies show a similar age-related decline of locomotor abilities. *P*-values are: 5-day-old flies: *P*_pEG>+ / EG>GFPdsRNA_=0.0014, *P*_pEG>+ / EG>rpr,hid_=0.0009, *P*_EG>GFPdsRNA / EG>rpr,hid_ >0.9999, 12-day-old flies: *P*_EG>GFPdsRNA / EG>hid_=0.0043. All other *P*-values are >0.05=non-significant (ns). Error bars indicate standard deviation. Quantification was done using a two-way ANOVA multiple comparison. (L) Longevity assay. 200 males and 200 virgin females of the genotypes indicated were kept on sugar only food. *rumpel* mutant males live 28% shorter than *w^1118^* control flies, *rumpel* mutant females live 8% shorter than *w^1118^* control flies (*P*_males_=2.43× e-34; *P*_females_=2×e-9).

RNA interference-based knockdown of *rumpel* caused paralysis of the adult flies upon mechanical stress (Schmidt et al., 2012). Since *rumpel* null mutant flies fail to show any of these responses and behaved as wild-type flies this initial observation is either due to off target effects or due to genetic plasticity induced upon systemic removal of the gene (Rossi et al., 2015; Sztal and Stainier, 2020). To better quantify behavioral phenotypes, we turned to larval locomotion. Third instar larvae of mutant *rumpel^C40^* showed slight differences, when comparing unconstrained locomotion at 25°C and at 32°C. A heat map representation of control and *rumpel* mutant larvae shows that at 25°C both control as well as *rumpel* mutants spread evenly across the tracking arena (Fig. 4A,B). In contrast at 32°C, *rumpel^C40^* null mutants do not explore the tracking plate as intensively as control larvae (Fig. 4C,D). This reduced exploratory locomotion phenotype is reflected in the mean distance to origin of the mutant animals (Fig. 4E, *n*=150 larvae, 3 min tracking, *P*=0.023**)**. Interestingly, when we tested *rumpel*^Δ*+cherry*^, that was backcrossed ten times against a *w^1118^* background, we noted no significant change in distance to origin at elevated temperatures (Fig. S3).

Ensheathing glial cells have been associated with sleep phenotypes (Davla et al., 2020; Stahl et al., 2018). Thus, we tested whether *rumpel*^Δ*+cherry*^ null mutants show an abnormal sleep behavior. We noted a significant increase in day sleep compared to *w^1118^* flies, similar to what was observed for taurin transporter (EAAT2) (Davla et al., 2020; Stahl et al., 2018) (Fig. 4F,G).

Rumpel is specifically expressed by ensheathing glia, therefore we tested the behavioral phenotypes of animals lacking ensheathing glia. Following expression of the pro-apoptotic genes *reaper* and *hid* specifically in ensheathing glia, these cells die, and adult flies survive with a median longevity of 32 days instead of 53 days (Pogodalla et al., 2021). To test whether flies lacking ensheathing glial cells are susceptible to temperature shock we treated 5-day-old male and mated female flies for 2 min at 40°C in a water bath. Wild-type Canton S flies as well as flies expressing *GFP^dsRNA^* recovered very quickly, and after 1 min, all flies were moving again (Fig. 4H). Flies lacking ensheathing glia remained paralyzed for several minutes, resembling the *rumpel* RNAi knockdown phenotype. Even after 4 min, 20% of the ensheathing glia ablated flies remained paralyzed (Fig. 4H). When we compared sleeping behavior of *rumpel* mutants and ensheathing glial ablated flies we noted a similarly significant increase of daytime sleep in both genotypes (Fig. 4F,G,I,J). To test whether adult locomotor ability is generally affected, we also performed a rapid iterative negative geotaxis assay (RING assay) (Gargano et al., 2005). We separately analyzed 5-, 12-, and 19-day-old females. 5-day-old control flies harboring only the split Gal4 construct performed slightly better when compared to flies that express an GFP^dsRNA^ (Fig. 4K). Comparing flies expressing *GFP^dsRNA^* in ensheathing glia with those that lack ensheathing glia behave similar. Likewise, we noted similar locomotor abilities in aged flies. indicating that ensheathing glial cells do not equally affect all adult locomotor behavior (Fig. 4K).

Taking the differential phenotypes of the different *rumpel* mutants, RNAi experiments and the results of the ablation experiments into account, we propose that genetic redundancy might explain the different phenotypic expressivity.

### Rumpel and Bumpel share similar expression patterns

Single cell RNAseq data (Davie et al., 2018) suggested that *rumpel* and *bumpel* are expressed by overlapping sets of glial cells, whereas *kumpel* expression appears restricted to the ensheathing glia (Fig. 1I–K). In addition, *in situ* hybridizations performed by the Berkeley genome project (Tomancak et al., 2002) showed also almost identical expression patterns of *bumpel* and *rumpel* during embryonic development (Fig. S4).

**Fig. 5.**
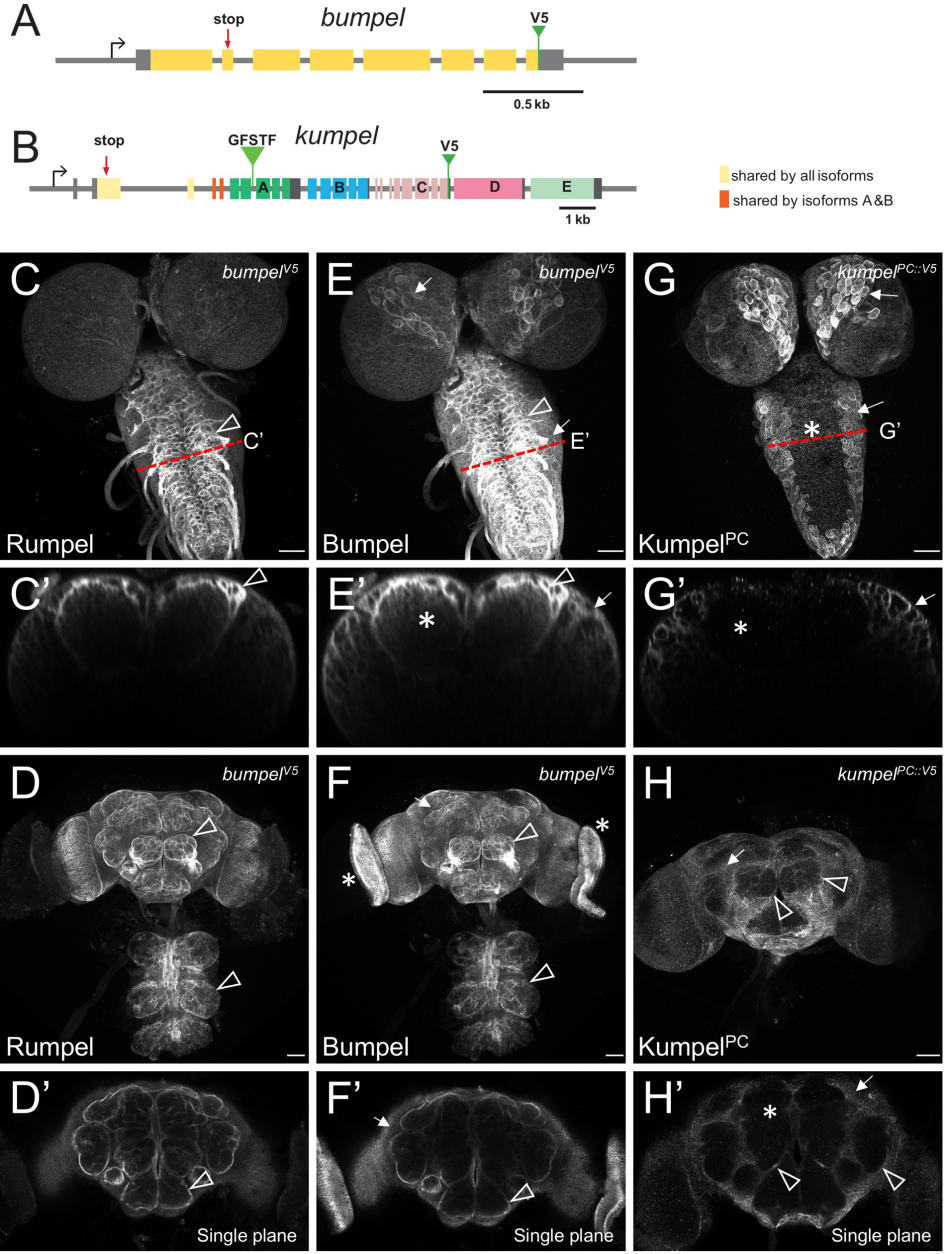
**Bumpel and Kumpel are both expressed in CNS glial cells.** (A,B) Schematic representation of the genomic loci of *bumpel* (*CG6723*) and *kumpel* (*CG42235*). Transcription is from left to right, coding exons are colored, five different isoforms are generated from the *kumpel* gene. The position of the stop codon mutations and the endogenously integrated V5 tags are indicated. GFSTF indicates the position of a MiMIC insertion. (C–H) Confocal analysis of third instar larval brains and adult brains stained for Rumpel, Bumpel^V5^ and Kumpel^PC::V5^ as indicated. Red dashed lines indicate the position of orthogonal planes shown in C′,E′,G′. (C,C′) Rumpel localizes predominantly in the ensheathing glial cells (arrowhead). (D) Maximum projections and (D′) single focal plane showing Rumpel localization in the adult brain. Rumpel is enriched in ensheathing glia (arrowheads). (E,E′) Bumpel^V5^ localizes to ensheathing glia (arrowhead) and cortex glial cells (arrows). Additional expression is noted in the neuropil (asterisk). (F,F′) In the adult nervous system, Bumpel localizes as detected for Rumpel. In addition, Bumpel^V5^ is found in the developing eyes (asterisk). (G,G′) Kumpel^PC::V5^ localizes predominantly to cortex glial cells (arrows). No Kumpel^PC::V5^ can be detected in the neuropil (asterisk). (H,H′) Kumpel^PC::V5^ localizes to the cortex glial cells in the adult brain (arrows). Only weak expression in adult ensheathing glia is noted (arrowhead, H). No Kumpel^PC::V5^ can be detected in the neuropil (asterisk).

To determine the protein localization of Bumpel, we inserted DNA sequences encoding a V5 tag at the 3′ end of the Bumpel coding region using a CRISPR-aided homologous recombination approach (see Materials and Methods for details, Fig. 5A). Homozygous *bumpel^V5^* flies eclosed in the expected Mendelian numbers and no abnormal phenotypes were detected. Endogenously tagged Bumpel protein is localized in a very similar pattern in the larval and adult brain, as observed for Rumpel (Fig. 5C–F). However, localization of Bumpel in cortex glial cells appeared slightly more pronounced (Fig. 5C,E). We also generated a *bumpel* minigene. *bumpel* is closely flanked by the genes *CG45676* and *Ipo9*. We cloned the entire *bumpel* gene locus including all flanking DNA sequences and the untranslated regions of *CG45676* and *Ipo9* and inserted a V5 tag at the C-terminus. The construct was placed on the second chromosome using the landing site 44F (Bischof et al., 2007) (Fig. S5A). In third instar larval brains, this construct also directs expression of Bumpel^V5^ in ensheathing and cortex glia (Fig. S5B,C).

### Kumpel expression in glial cells

In contrast to *rumpel* and *bumpel*, *kumpel* has a complex genomic organization and differential splicing is expected to generate five distinct isoforms (Kumpel^PA-PE^, Fig. 5B). Only the first two exons, which encode the N-terminal 236 amino acids are shared by all isoforms. These two exons encode the signal sequence and the first 1.5 of 13 predicted transmembrane domains. Two isoforms (PA and PB) share another two exons that encode a further 1.5 transmembrane domains. All other protein parts are unique to the different isoforms and given the conserved exon-intron arrangement, appear to originate from an ancient gene duplication.

To determine the *kumpel* expression pattern we inserted a V5 tag into the endogenous gene locus at the 3′ end of the last Kumpel^PC^ encoding exon (Fig. 5B). This isoform mostly localizes in cortex glial cells in the larval, as well as in the adult nervous system (Fig. 5G,H). Kumpel^PC^ appears to be weakly expressed by the adult ensheathing glia (Fig. 5H, arrowheads). Single cell sequencing data (Fig. 1K) indicates strongest expression of *kumpel* in ensheathing glial cells. Thus, other Kumpel isoforms may possibly show a more defined localization in ensheathing glial cells. To address their expression, we used an available converted MiMIC insertion line (*MI05542-GFSTS.0*), which directs expression of Kumpel^PA^-GFP fusion protein. However, this protein trap also labels mostly cortex glial cells (Fig. S5D,E).

### Generation of *bumpel* and *kumpel* mutants

To further study possible genetic relationships between *rumpel*, *bumpel* and *kumpel* we generated CRISPR induced mutants. The *bumpel* gene was targeted in first exon resulting in a frameshift at position +40 bp of the open reading frame, causing an early stop codon (Fig. 5A). The mutant is therefore predicted to be a null allele. Homozygous *bumpel* mutant flies eclosed at the expected Mendelian ratio and showed no fertility or morphological abnormalities. Adult flies also do not show any heat or bang sensitivity. Likewise, locomotion of *bumpel* mutant larvae is indistinguishable from the control at 25°C as well as at 32°C.

To induce *kumpel* mutants, we targeted the first common exon present in all *kumpel* transcripts (Fig. 5B). The mutation at position +253 bp of the reading frame caused a frameshift and subsequent termination of translation before the first transmembrane. The mutation can therefore be considered as null mutation. Mutant *kumpel* flies are homozygous viable and fertile and show no discernible abnormal phenotypes. As noted for *bumpel* mutant flies they show no locomotor deficits.

### *rumpel*, *bumpel* and *kumpel* genetically interact

**Fig. 6.**
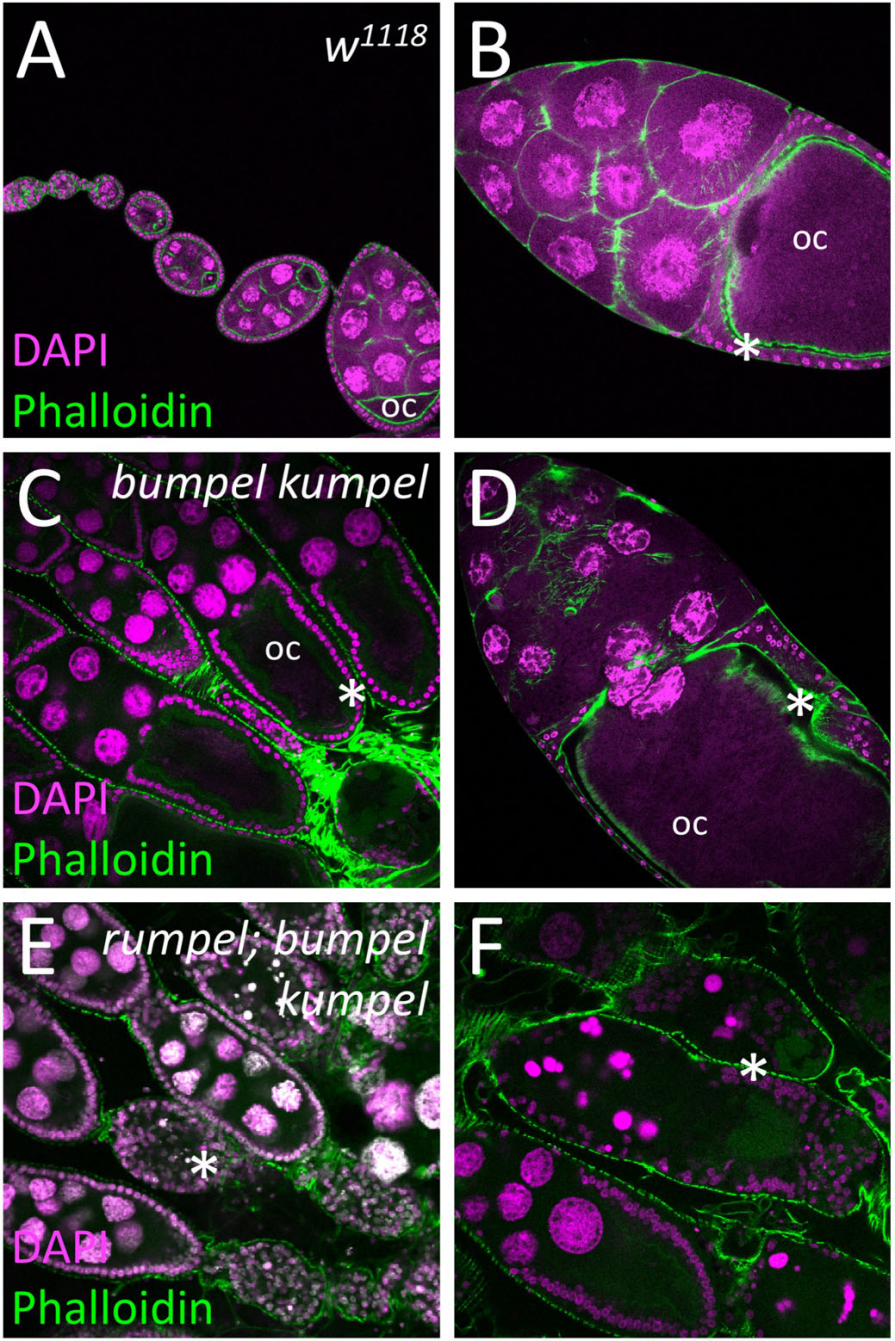
**SLC5A transporters are required for the oogenesis.** Confocal analysis of wild-type and mutant ovaries. Nuclei are labeled by DAPI staining, F-actin is shown following phalloidin staining (green). (A,B) In control females oogenesis developing egg chambers connected by stalk cells mature to form tubular ovarioles. During the previtellogenic phase, the future oocyte (oc) is defined which is positioned at the posterior pole. During the vitellogenic phases the oocyte grows exponentially and is surrounded by a cuboidal follicular epithelium (asterisks). (C,D) Homozygous *bumpel kumpel* double mutants are sterile but lay few eggs. Oogenesis is affected at the vitellogenic phase. The oocyte and the follicle epithelium degenerate. (E,F) Homozygous *rumpel bumpel kumpel* mutants are sterile and never lay eggs. Oogenesis is affected at the vitellogenic stage as seen in *rumpel bumpel* double mutants. However, the disintegration of oocytes and the follicular epithelium is more pronounced.

*rumpel*, *bumpel* and *kumpel* encode highly related proteins that show similar expression patterns. To further determine possible redundancy between the different gene functions we first generated double mutant combinations. *rumpel bumpel* or *rumpel kumpel* double mutants are viable and fertile. Larval locomotion of double mutants is as of the single mutants. *bumpel kumpel* double mutant flies are also viable. However, homozygous females show reduced egg laying and are sterile. In these double mutants, oogenesis initially proceeds normally until stage eight. However, during the subsequent vitellogenic phase oogenesis appears disrupted and no normal eggs are formed, and they cannot be fertilized (Fig. 6A–D). We next generated *rumpel bumpel kumpel* triple mutant flies. Triple mutant females are sterile and do not lay any eggs. In contrast to the *bumpel kumpel* double mutant, oogenesis appears completely blocked after stage eight (Fig. 6E,F).

We next tested whether the triple mutant shows a locomotor phenotype. At 25°C, *rumpel*^Δ*+cherry*^ mutant larvae have slightly reduced distance to origin after 3 min free crawling compared to control larvae (Fig. S3). A very similar reduction in the distance to origin is detected for the triple mutant (Fig. S3). In contrast, at elevated temperature (32°C) the triple mutant shows a significantly reduced distance to origin whereas control and *rumpel*^Δ*+cherry*^ mutants are not affected by the increase in temperature (Fig. S3). Further analysis of the different locomotion parameters revealed that although triple mutant larvae have a reduced distance to origin, they are faster, but show an altered bending behavior (Fig. S3).

### *rumpel*, *bumpel* and *kumpel* encode SLC5A homologs that likely transport lactate

The sterility phenotype shown by the double mutant animals allowed us to conduct rescue experiments. One copy of the *bumpel* minigene rescued fertility of the *bumpel kumpel* double mutant. Interestingly, overexpression of *bumpel* by introducing two copies of the *bumpel* minigene into a wild-type background but not in a heterozygous mutant background causes a lethal phenotype. Likewise, pan-glial *repo-Gal4* based overexpression of a *UAS-Bumpel^3xHA^* construct (Bischof et al., 2013) causes lethality. Rescue experiments using Gal4 based expression of the *kumpel^PD^* isoform resulted in a few larvae but did not rescue beyond larval stages. This suggests that expression levels are likely crucial for function.

The predicted Rumpel, Bumpel and Kumpel proteins all belong to the SLC5A family of monocarboxylate transporters that utilize a sodium gradient across the plasma membrane to transport a variety of solutes. To determine the nature of these solutes we utilized the knowledge on transported metabolites of the mammalian orthologues SLC5A1-SLC5A12. We obtained full-length cDNAs, encoding all transporters except SLC5A4 and SLC5A6 (Table 1) and generated transgenic flies expressing the different mammalian SLC5A proteins under UAS control. We generated females with the following genotypes (*Act5C-Gal4/UAS-SLC5Axy, bumpel kumpel / bumpel kumpel*) and assayed whether female sterility was rescued when crossed to CantonS males. To our surprise, almost all transgenes showed very limited rescue after prolonged culture (Table 1). Ubiquitous expression of SLC5A2, which transports glucose (Wright, 2013), gave robust rescue with many flies eclosing from homozygous *bumpel kumpel* mothers. Weaker rescue with low numbers of eclosing flies was noted following expression of SLC5A8 and SLC5A12 (which transport lactate; Coady et al., 2004; Gopal et al., 2007; Miyauchi et al., 2004) (Table 1).

**Table 1.**
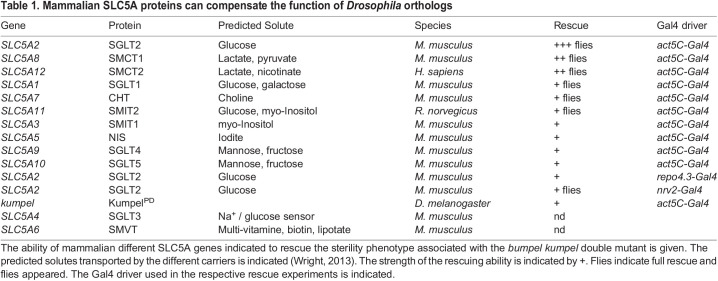
Mammalian SLC5A proteins can compensate the function of *Drosophila* orthologs

The above rescue data suggests that ubiquitously induced glucose and/or lactate transport is able to rescue the *kumpel bumpel* double mutant sterility phenotype. To address the question whether glial expression is sufficient for rescue, we expressed SLC5A2, SLC5A8 and SLC5A12 using *repo-Gal4* and *nrv2-Gal4*. Rescue using *nrv2-Gal4* directed expression, (*nrv2* being expressed in many glial cells and very weakly in the ovary; Cash and Andrews, 2012; Graveley et al., 2011) produced a small number of surviving flies. Interestingly, exclusive panglial expression resulted in few, small larvae, which indicate rescue; however, no flies eclosed.

## DISCUSSION

Here we describe the analysis of three predicted *Drosophila* solute carrier proteins: Rumpel, Bumpel and Kumpel, which show overlapping expression patterns in the larval and the adult CNS. The *rumpel* gene was initially identified in an RNAi-based screen for adult locomotor defects (Schmidt et al., 2012). The same behavioral phenotype was also found in a different study but unlike our findings the phenotype was assigned to defects in astrocyte-like glial cells (Ng and Jackson, 2015). We thus generated mutants and surprisingly, detected only very weak behavioral phenotypes. The notion that the locomotor phenotype was stronger in animals carrying a CRISPR induced point mutant compared to animals carrying a deletion of the *rumpel* locus could possibly indicate the presence of a nonsense mediated decay mechanism that leads to a more global change of the transcriptional activity of the cell. This was also corroborated by the finding that flies lacking all ensheathing glia show a pronounced heat shock sensitivity.

To determine whether a possible genetic redundancy is causing this apparent lack of abnormal phenotypes in *rumpel* mutants, we analyzed the most closely related genes *bumpel* and *kumpel*. However, even triple mutants do not show the initially observed RNAi-induced locomotor phenotype, though we did find a prominent reduction in exploratory locomotion and thus a reduced distance to origin. A genetic redundancy, however, is detected during oogenesis. In contrast to single mutants, in *bumpel kumpel* double mutants, oogenesis is defective during the vitellogenic phase and in *rumpel bumpel kumpel* triple mutant females oogenesis arrests shortly before the vitellogenic phase.

In *Drosophila*, the complexity of the SLC5A family transporters is similar to the one found in mammals and 14 SLC5A proteins are encoded in the fly genome (Featherstone, 2011). Only three of these genes have been analyzed in greater detail. The *Sodium-dependent multivitamin transporter* (*Smvt*) is not expressed in the CNS, but its muscle specific knockdown causes a flightless phenotype (Schnorrer et al., 2010). The gene *salty dog* (*salt*) is also not expressed in the CNS and affects survival in a high-salt environment (Stergiopoulos et al., 2009). The gene *cupcake* (*SLC5A11*) is prominently expressed in neurons of the adult fly brain where it is required for proper food selection (Dus et al., 2013). Here, we present analysis of three additional members of the SLC5A family which not only share protein homology but also show overlapping expression patterns. All single mutants are viable and fertile with no obvious behavioral phenotypes.

Similar to *rumpel*, where a RNAi mediated knockdown causes a phenotype but not the *rumpel* mutant, *Mfs3* and *pippin* knockdown but not *Mfs3* and *pippin* mutants show a compensatory upregulation of Tret1-1. Interestingly, *Mfs3*, *pippin* and *Tret1-1* all encode carbohydrate transporters. Which mechanisms could account for such effects? Most easily they could be explained as off-target effects, however, no such off-targets are predicted for the RNAi strain used (Dietzl et al., 2007). An alternative explanation is that the construct directing expression of double RNA is inserted in gene that dominantly contributes to the phenotype. However, it is also possible that this is a result of a more general transcriptional adaptation (Sztal and Stainier, 2020). First identified in zebrafish while studying the role of the endothelial extracellular matrix (ECM) protein Egfl7 (Rossi et al., 2015). Morpholino-based knockdown of the gene encoding this protein resulted in a severe phenotype in zebrafish (as well as in Xenopus or in human cells) but the corresponding mutant appeared largely normal. Subsequent mass spectrometry revealed the upregulation of the related ECM protein Emilin3a in the *Egfl7* mutant but not in the knockdown animal. Subsequently it was shown that mutant mRNA degradation plays a crucial role in activating transcriptional adaptation in zebrafish and mouse cell lines and a clean deletion of the gene is not sufficient to trigger transcriptional adaptation (El-Brolosy et al., 2019). However, in contrast to what is observed for zebrafish, *rumpel* excision mutants that lack all *rumpel* transcripts still exhibit no abnormal phenotype.

Here we show that Rumpel, Bumpel and Kumpel are all expressed in overlapping sets of CNS glial cells. In addition, RNA-seq data obtained from dissected tissues show no expression of *rumpel*, *bumpel* or *kumpel* in ovaries (Graveley et al., 2011). Yet, the double and triple mutants are sterile with an ovary phenotype, which allowed us to perform rescue experiments using an array of heterologous SLC5A transporters with defined solute transport properties. As control, we performed rescue using the *bumpel^minigene^* construct. Homozygous *bumpel^minigene^* animals are lethal and this lethality is rescued by the *bumpel kumpel* double mutant. Likewise, the sterility phenotype associated with homozygous *bumpel kumpel* double mutants is rescued by a single copy of the *bumpel^minigene^*. Thus, the expression level of *bumpel* appears tightly regulated, and an overexpression of SLC5A is more detrimental than a complete lack of SLC5A transporter. This may have obscured the rescue experiments using heterologous SLC5A sequences, which indicated that Bumpel and/or Kumpel transport glucose or lactate.

The *Drosophila* adult ovary comprises a pair of 16–20 tubular ovarioles, where egg chambers connected by stalk cells mature in a sequential manner (Margolis and Spradling, 1995; Robinson and Cooley, 1997; Spradling, 1993). Two phases of egg chamber growth can be defined: the previtellogenic and the vitellogenic phases. During the vitellogenic phase an exponential l growth of the oocyte occurs due to yolk import through the follicular cells surrounding the egg chamber. The onset of the vitellogenic phase is controlled by hormones and the nutritional state of the fly, which is generally regulated by insulin-like peptides (Mendes and Mirth, 2016; Mirth et al., 2014; Nässel and Vanden Broeck, 2016; Raushenbach et al., 2004; Richard et al., 2005). The *rumpel bumpel kumpel* triple mutant specifically affects the vitellogenic phase, suggesting that glucose metabolism in the brain controls ovary development. Such a brain-gonad axis had been described before. For example, the neurotransmitter octopamine, which is closely related to norepinephrine, is known to act as an alerting signal in insects and octopaminergic neurons reach almost all peripheral tissues (Pauls et al., 2018). The endocytic regulator monensin-sensitive 1 (Mon1) is required in octopaminergic neurons for normal ovary growth and a cell-type specific knockdown results in the absence of late-stage egg chambers (Dhiman et al., 2019). Octopamine also reaches the insulin producing cells (IPCs), which are known to regulate feeding behavior and express the octopamine receptor OAMB1 (Luo et al., 2014; Selcho and Pauls, 2019). It is possible, disruption of SLC5A function in the *Drosophila* glial cells affects nutrient sensing in the nervous system, which feeds back to octopaminergic neurons, and thereby hinders ovarian development.

## MATERIALS AND METHODS

### *Drosophila* work

Unless otherwise stated, all *Drosophila* stocks and crosses were raised on standard *Drosophila* food at 25°C. To target all glial cells we employed *repo4.3-Gal4* (Schmidt et al., 2012), to specifically target glial subsets we used *83E12-Gal4* (ensheathing glia) (Li et al., 2014; Otto et al., 2018)*, nrv2-Gal4* (cortex and ensheathing glia) (Sun et al., 1999), *R55B12-Gal4* (cortex glia) (Li et al., 2014), *alrm-Gal4* (astrocyte-like glia) (Muthukumar et al., 2014). UAS-dsRNA lines targeting *rumpel* (GD3270, KK106220), were obtained from the VDRC (Vienna, Austria). *UAS-GFP^dsRNA^* (BL9330, BL9331), *kumpel^MiMIC-GFSTF^* (BL60231), *UAS-lam::GFP* (BL7378), *act5C-Gal4* (BL4414), *UAS-rpr.C* (BL5823), *UAS-hid.Z* (BL65403), and *Canton S* (BL64309) were obtained from the Bloomington stock center (Bloomington, Indiana, USA). *UAS-bumpel* (F003123) was obtained from the FlyORF collection (Zürich, Switzerland).

### Generation of mutants and transgenes

All single-guide RNA (sgRNA) and PCR primers used in this study are listed below in Table 2. To generate mutants, sgRNA plasmids were injected into Cas9 expressing recipient embryos (Port et al., 2014). Indels were detected in the F1 generation using PCR and subsequent sequence analysis. To replace the *rumpel* locus with mCherry, we generated a donor plasmid starting from pTV3 (kindly provided by J.P. Vincent, London) where mCherry is flanked by 2 kb flanking genomic rumpel DNA. To generate endogenous V5-tags, we generated donor plasmids where the V5 tag is inserted just before the stop codon, flanked by 1.5 kb of genomic sequence on either side. The *bumpel^V5^* minigene spans ∼3.1 kb of genomic DNA, including the UTRs of the neighboring genes. We inserted V5 tag just before the stop codon using standard procedures. The construct was inserted into the *44F* landing site (Bischof et al., 2007). The different mutants and transgenes generated in this study are listed in Table 3.

**Table 2.**
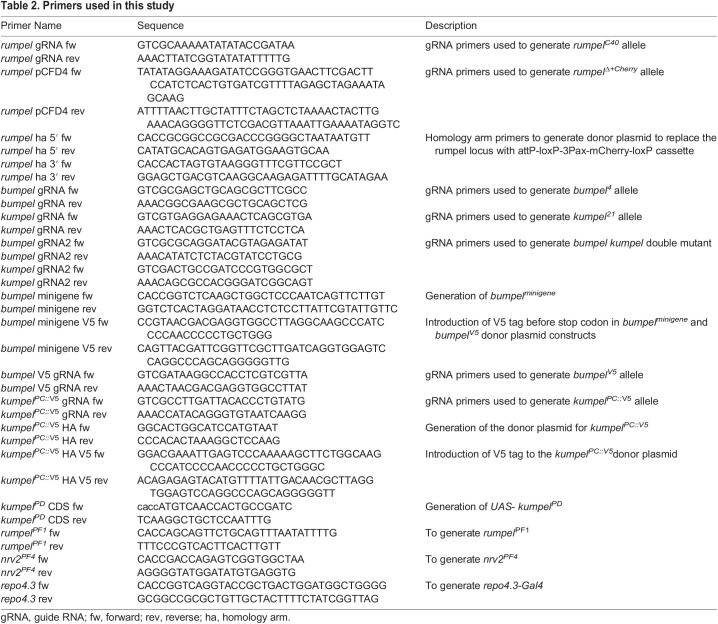
Primers used in this study

**Table 3.**
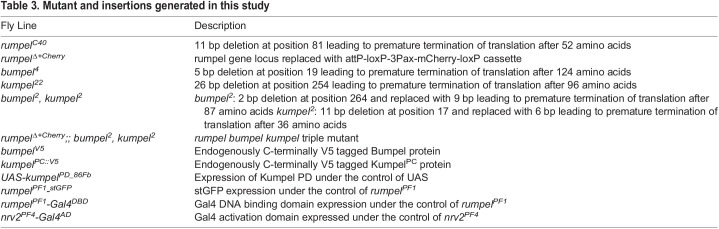
Mutant and insertions generated in this study

### Generation of Interspecies rescue construct

To generate interspecies rescue constructs, we amplified the open reading frames of the mammalian *SLC5A* genes from their respective cDNA plasmids, which were obtained from Thermo Fisher Scientific (see Table 4). We generated UAS-based expression plasmids using *pUAST-attB-rfa* and inserted them into *51C^RFP+^* landing site.

**Table 4.**
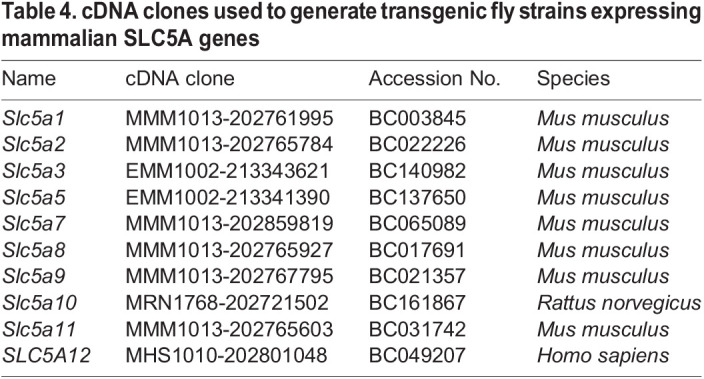
cDNA clones used to generate transgenic fly strains expressing mammalian SLC5A genes

For the interspecies rescue experiments, we have recombined *Act5C-Gal4* with *UAS-SLC5Axy* and then generated *Act5C-Gal4*, *UAS-SLC5Axy; bumpel^2^, kumpel^2^*. Generated homozygous females were crossed with wild-type CantonS males. The fertility rescue was scored as larval, pupal, full rescue or no rescue in the F1 generation. Furthermore, *UAS-SLC5A2* was recombined with *repo4.3-Gal4* and *nrv2-Gal4*. Glial interspecies rescue was performed as explained above.

### Longevity assay

Virgin female and male flies (*n*=200) were kept in groups of 20 at 25°C and were transferred to fresh food three times a week. Dead flies were then counted. Agar with 5% sucrose was used as food source. Survival rates were determined using the Kaplan–Meier approach and calculated *P*-values using Log Rank test.

### Behavioral analyses

Larval behavioral experiments were performed at 25°C unless otherwise indicated. Larval locomotion was analyzed using FIM (Risse et al., 2017, 2013). Locomotion of 10–15 larvae was recorded for 3 min at ten frames per second. Tracking data was analyzed as described (Otto et al., 2018, 2016; Risse et al., 2014). Distance to origin is defined by the distance of the larva from the spot where it placed on the agar plate, normalized per minute. In bending distribution plots, the number of head bends per 10 s deviating from the larval 180° body axis are shown. Velocity during Go-phases is defined as distance per time (pixels per second) during larval go phases.

To obtain a heatmap representation of larval distribution on the tracking area we employed the open source RStudio software (http://www.rstudio.com). In a custom-made script, the tracking area was divided into 50×50 px squares, which is in the same size range as the average larval length (45 px in these settings). The frequency of an appearance of a larva in each square was calculated and is indicated by shading intensity. In all experiments, the same number of larvae in the same area over the same length. For the analysis of the sleep phenotype, freshly hatched males were collected and aged at 25°C for 3 days. 40 single 3-day-old male flies placed into individual capillaries mounted in an Ethoscope arena were analyzed as described (Geissmann et al., 2017). Here individual flies are constantly video-tracked for 7 days at constant temperature (25°C) and humidity (65%) with 12 h light-dark cycle. Sleep was defined as 5 min with no activity and was quantified using the Rhetomics package in R (Geissmann et al., 2019). For statistical analyses the standard non-parametrical Wilcoxon rank-sum test was performed using FIM Analytics 2 or R.

To test for temperature sensitivity, 3–5-day-old flies staged males and females were collected and five flies each were placed into a new vial. On the next day, the five flies were transferred into a fresh empty vial without food and anesthesia. After a 5–10 min acclimation time the vials were placed in a water bath at 40°C for 2 min. Afterwards, the vials were filmed for 4 min at room temperature. Flies, which lay on their backs and did not move, were counted as paralyzed. 100 flies for each genotype were recorded.

To determine the negative geotaxis, 100 females were separately collected and staged as required. Ten flies each were placed in fresh vials with standard food and kept overnight at 25°C. Afterwards, the ten flies were loaded in long plastic tubes without anesthesia. After 5–10 min acclimation time the tubes were placed in the rapid iterative negative geotaxis (RING) system according to Gargano et al. (2005). In total, 100 flies were tested. The images were processed using Fiji with MTrack3_.jar plugin and AutoRun2.ijm macro. The mean velocity was determined using the RING assay Script.R in the R program. Statistical analysis was performed by Prism 6.0.

### Immunohistochemistry

Immunohistochemistry was performed according to standard protocols except the fixation process. Larvae were dissected in ice-cold PBS. Filet preparations were fixed in Bouin's Solution (Sigma-Aldrich) for 3 min at room temperature, while the adult brains were fixed in 2% PFA for 90 min at room temperature. The following antibodies were used: mouse anti-Repo (DSHB, 1:5), rat anti-DN-Cadherin (DSHB, 1:10), mouse anti-V5 (Thermo Fisher Scientific, 1:500), rabbit anti-GFP (Thermo Fisher Scientific, 1:1000), rabbit anti-dsRed (Clontech, 1:1000), goat anti-HRP-Cy5 (Dianova, 1:200). Rabbit anti-Rumpel peptide antibodies were generated against the C-terminal domain of Rumpel (Pineda, Berlin, 1:500). Secondary antibodies conjugated to Alexa Fluor 488, Alexa Fluor 568 or Alexa Fluor 647 were used (Thermo Fisher Scientific, 1:1000). Ovaries were dissected in ice-cold PBS and fixed in 4% PFA for 15 min. Immunohistochemistry for ovaries was performed as described (Bogdan et al., 2005). Alexa Fluor 568 Phalloidin (Thermo Fisher Scientific, 1:100) and DAPI (Thermo Fisher Scientific, 1:1000). Confocal microscopy data was generated using a Zeiss 710 or 880 LSM or Leica TCS SP8 DLS. Images were acquired using either the Zeiss LSM ZEN imaging software, or the LSM LAS X software and analyzed using Fiji (Schindelin et al., 2012).

### Paralogs and orthologs of *rumpel*

The amino acid sequences of Rumpel and its closest mouse orthologues SLC5A5, SLC5A8 and SLC5A9 were aligned and visualized using T-Coffee tool (http://tcoffee.crg.cat/apps/tcoffee/do:regular) and Boxshade (https://embnet.vital-it.ch/software/BOX_form.html). For amino acid sequence comparisons, we used Clustal Ω (https://www.ebi.ac.uk/Tools/msa/clustalo/) and Blast (https://blast.ncbi.nlm.nih.gov). To reconstruct the phylogenetic tree of the *rumpel* paralogs we employed MEGA X (default settings; Kumar et al., 2018; Stecher et al., 2020).

## Supplementary Material

Supplementary information
