# Supplementary material for: Redundant functions of the SLC5A transporters Rumpel, Bumpel, and Kumpel in ensheathing glial cells
Source: Biol Open. 2022 Jan 18;11(1):bio059128. doi: 10.1242/bio.059128 (PMC8790523; doi:10.1242/bio.059128)
Supplement: Supplementary information [file biolopen-11-059128-s1.pdf]

```
Rumpel      1 MHVEELRASLQS-F--------GWPDYLVFCLMLAICAVIGIYFCLQLRLESRKQKANGS
mSLC5A2     1 MEQHV---EAGSELGEQKVLIDNPADILVIAAYFLLVIGVGLWSMFRTN-----------
mSLC5A8     1 MDASR---DIGS-F--------VVWDYVVFAGMLLISAAIGIYYAFAGG-----------
mSLC5A12    1 MRV-------KN-F--------EAWDYVVFAGLFVISSGIGVFFAIKER-----------

Rumpel     52 EEAASSAASYLVGGRQMKIFPITMSLISSFISGITLLGTPTEVYLYGAQYMYIMGSLVLM
mSLC5A2    47 --R-GTVGGYFLAGRSMVWWPVGASLFASNIGSGHFVGLAGTGAASGLAVAGFEWNALFV
mSLC5A8    38 --GQQTSKDFLMGGRSMSAVPVALSLTASFMSAVTVLGTPAEVYRFGAIFSIFVITYFFV
mSLC5A12   34 --KKTTSREFLVGGRQMSFGPVALSLTASFMSAVTVLGTPAEVYRFGASFFLFLISYVFV

Rumpel    112 GFCMYYFFLPVFHELNLISTYKYLEQRYNR-SLRLFGSVMFIVASLL-WLPIVIYVPAIA
mSLC5A2   104 VLLLGWLFVPVYLTAGVITMPQYLRKRFGGHRIRLYLSVLSLFLYIFTKISVDMFSGAVF
mSLC5A8    96 VVISAEVFLPVFYRLGITSTYEYLELRFNR-CIRLCGTILFIVQTIL-YTGIVIYAPALA
mSLC5A12   92 VFFTSELFLPVFYRSGITSTYEYLQLRFNK-PVRYAATIIYIVQTIL-YTGVVVYAPALA

Rumpel    170 FNQATGVNIHIVTPIVCVVCIFYTCIGGLKAVVWTDVIQTLIMFGAMALVLIKGTLDIGG
mSLC5A2   164 IQQALGWNIYASVIALLGITMIYTVTGGLAALMYTDTVQTFVILAGAFILTGYAFHEVGG
mSLC5A8   154 LNQVTGFDLWGAVVATGVVCTFYCTLGGLKAVVWTDVFQVGIMVAGFASVIIQASITQHG
mSLC5A12  150 LNQVTGFNLWASVFATGIVCTFYCSLGGLKAVVWTDAFQMVVMIVGFLTVLIQGSNHVGG

Rumpel    230 PGVVWQKAQET------------ARLEVPNFSA---------DLSERYTFYSLV-LGGVA
mSLC5A2   224 YSGLFDKYLGAMTSLTVSKDPSVGNISSTCYQPRPDSYHLLRDPVTGDLPWPALLLGLTI
mSLC5A8   214 INKILSDAFNG------------GRLNFWNFDP---------NPLQRHTFWTIV-IGGTF
mSLC5A12  210 FNNVLEKAGNG------------SRLHIVDFDV---------DPLRRHTFWTIT-IGGTF

Rumpel    268 HWLKSNAISQNMIQRYLSLPTLRDARIA----IWTFIAGVLAFLMICGYTGLLIYATYSQ
mSLC5A2   284 VSGWYWCSDQVIVQRCLAGKNLTHIKAGCILCGYLKLMPMFLMVMPGMI-SRILYPDEVA
mSLC5A8   252 TWTTIYGVNQSQVQRYISCKSRLHAKLS----LYVNLVGLWVILTCSIFCGLALYSRYRE
mSLC5A12  248 TWLGVYGVNQSTIQRCISCKTEKHAKLA----LYFNLLGLWIIVACAVFSGLIMYSHFKD

Rumpel    324 CD------PLETKLAQRNDQLLPLLVMDTLGSYPGLPGVFVAGVFSAALSSLSTGLNSLS
mSLC5A2   343 CVVPEVCKRVCGTEVGCSNIAYPRLVVKLMP--NGLRGLMLAVMLAALMSSLASIFNSSS
mSLC5A8   308 CD------PWTSKKVSAIDQLMPYLVLDILKNYPGVPGLFVACAYSGTLSTVSSSINALA
mSLC5A12  304 CD------PWTSGVISAPDQLMPYFVMEIFATMPGLPGLFVACAFSGTLSTVAASINALA

Rumpel    378 AVVLEDFVKTFRKSPLTEGQTAFVMRSVVVVFGIIFVALV--FAVEKLGAVLQLTITLSS
mSLC5A2   401 TLFTMDIYTRLR-PRAGDKELLLVGRLWVVFIVAVSVAWLPVVQAAQGGQLFDYIQSVSS
mSLC5A8   362 AVTVEDLIKPRF-KSLSEKSLSWISQGMSVLYGALCIGMA--ALASLMGALLQAALSIFG
mSLC5A12  358 TVTFEDFVKSCF-PHLSDKLSTWISKGLCILFGIMCTSMA--VVASLMGSVVQAALSIHG

Rumpel    436 VANGPLLGIFTAGVMLPWVNSKGALLGGFSSLIVMAWMCVSAQRDLVTGDLVYKRKPYST
mSLC5A2   460 YLAPPVSAVFVLALFVPRVNEKGAFWGLVGGLLMGLARLIPEFFFG-SGS----------
mSLC5A8   419 MVGGPLLGLFSLGILVPFANSIGALTGLLAGFAISLWVGIGAQLYPPLPERT-LPLPLET
mSLC5A12  415 MCGGPMLGLFTLGLVFPFVNWKGALGGLLTGITLSFWVAIGSFIYPAPESKT-LPLPLST

Rumpel    496 MGCNYTFAGEPRE------FASLALDGAI--SSVPSA-PFQLYRISYLYFTLFGALVTIV
mSLC5A2   509 -------------------------------CVRPSACPALFCRVHYLYFAIILFICSGI
mSLC5A8   478 YGCNITHNGSDWMSTTEMPFSTS--AFQIHNAERTPL-MDNWYSLSYLYFSTIGTLTTLF
mSLC5A12  474 EHCVEL-----NITT------TV--APQI--SSRPVL-ADTWYSLSYLYFSAVGCLGCIA

Rumpel    547 VALVTSLLLRETDLDGVDTRLLTPFVRRWL-----------ERRRH---RRS--------
mSLC5A2   538 LTLGISLCTAPIPQKHLHR-LVF-------------------SLRHSKEERED-LDADE-
mSLC5A8   535 VGILISLSTGG-RKQNLDPRFLL--------------------------TKQDFLSNFDV
mSLC5A12  518 AGIIISFLTGKQRGKDIDPLLIRPVCNLFCFWSKKYKTLCWCGVQHDRETEQDYLDSGSA

Rumpel    585 -------------KIPDSGQK-----------S--SN--------KQE---------TKT
mSLC5A2   576 LEGP------APAPVQNGGQECAMEMEEVQSPAPGLLRRCLLWFCGMSKSGSGSPP-PTT
mSLC5A8   568 FKKRNHVLNYKLHPVEVGGTD-----------NPAFN--------HVELNFTDHSGKING
mSLC5A12  578 WKQG------VESGLQNGLKQ-----DT-LAQIPGYN--------PKEKSYSNSVP-EKT

Rumpel    602 ------------------------------------------
mSLC5A2   629 EEVAATTRRLEDISEDPRWARVVNLNALLMMTVAVFLWGFYA
mSLC5A8   609 TRL---------------------------------------
mSLC5A12  617 TYF---------------------------------------
```

Biology Open • Supplementary information

Fig. S1. Rumpel is predicted to be a member of the Sodium Solute symporter family (SLC5)

BLAST detects highest homology with SLC5A8 (35.77 % identity with 90% coverage, p=5e-112) and SLC5A12 (33.33 % identity with 92% coverage p= 3e-110). Less significant homology detected is SLC5A2 (23.21% with 67% coverage identity, p=3e-12). Identical and similar amino acids among proteins are indicated as black and grey fills, respectively. “-” indicates missing amino acids in the respective sequence.

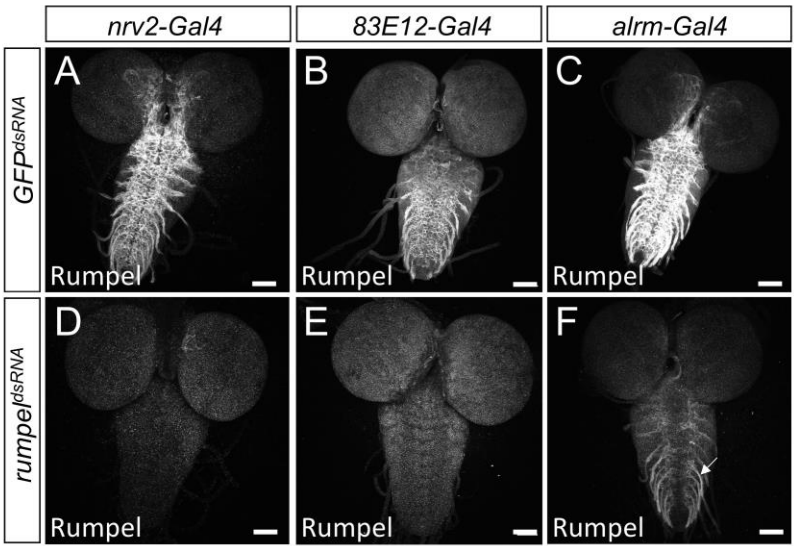

Fig. S2. Rumpel is expressed predominantly by ensheathing glial cells.

All specimens are stained for Rumpel (grey). (A-F) Confocal maximum projections of a third instar larval brain stained for Rumpel. (A-C) Rumpel expression is not affected by expressing dsRNA directed against *GFP* (*nrv2-Gal4>GFP*^{dsRNABL9330}; A), in ensheathing glia (*83E12-Gal4> GFP*^{dsRNABL9331}; B) or astrocyte-like glial cells (*alrm-Gal4>GFP*^{dsRNABL9330}; C). (D-F) No Rumpel expression is detected upon expression of dsRNA directed against *rumpel* in cortex glia and ensheathing glia (*nrv2-Gal4> rumpel*^{dsRNAv43922}; D). Following suppression of rumpel expression in ensheathing glial cells faint expression can be noted in the cortex and in the neuropil (*83E12-Gal4>rumpel*^{dsRNAv107361}; E). Upon silencing of rumpel only in astrocyte-like glial cells (*alrm-Gal4>rumpel*^{dsRNAv43922}; F) weak Rumpel expression can be detected in ensheathing glial cells (arrow) and in the cortex. Scale bars are 50 µm.

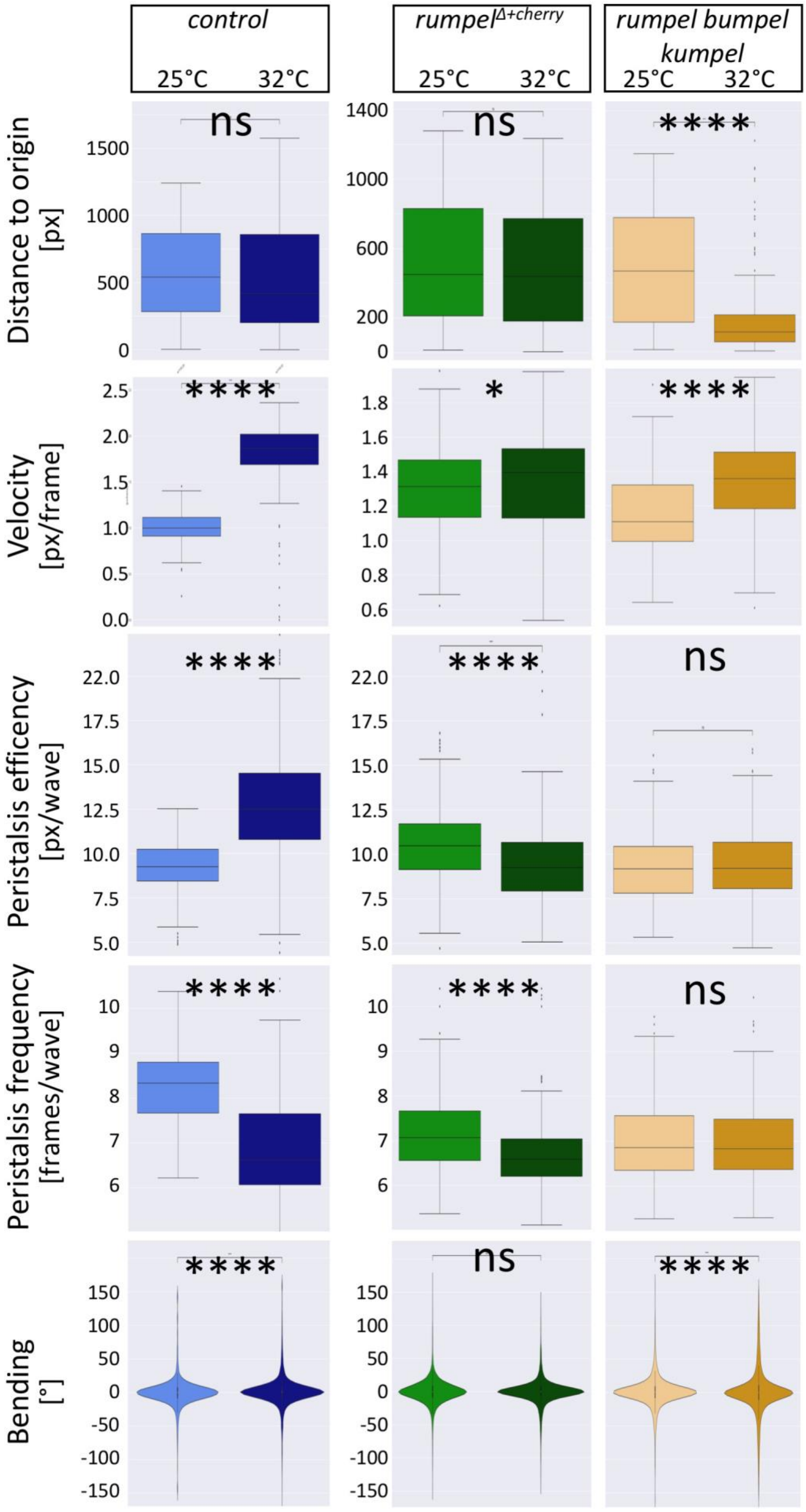

Biology Open • Supplementary information

Fig. S3. ***rumpel bumpel kumpel*** **triple mutant show a subtle temperature dependent locomotion phenotype**

Free locomotion of 3-4 groups of 15 third instar larvae of the following genotypes [*rumpel*$^{\Delta+cherry}$, *rumpel*$^{\Delta+cherry}$ *bumpel*^{2} *kumpel*^{2}, *w*^{1118}]. Although crawling velocity increases, control larvae [*w*^{1118}] show no reduced distance to origin at 32°C compared to 25°C, which is likely due to increased bending behavior. In *rumpel* mutant larvae, no difference in distance to origin can be determined. Locomotion speed increases very slightly which is due to faster not as efficient peristaltic contractions. In *rumpel bumpel kumpel* triple mutants distance to origin is dramatically affected, despite the fact that velocity increases by 20% due to an increased bending behavior.

Biology Open • Supplementary information

A

Percent amino acid identity

**Proteins**	**CG9657 Rumpel**	**CG6723 Bumpel**	**CG42235 Kumpel^{PA}**	**CG42235 Kumpel^{PB}**	**CG42235 Kumpel^{PC}**	**CG42235 Kumpel^{PD}**	**CG42235 Kumpel^{PE}**
**Rumpel**	-	51.61%	47.94%	47.94%	46.40%	43.04%	46.54%
**Bumpel**	52.55%	-	48.03%	46.68%	43.26%	43.43%	43.64%
**Kumpel^{PA}**	49.48%	48.03%	-	85.88%	63.83%	62.30%	64.01%
**Kumpel^{PB}**	49.14%	46.68%	85.88%	-	64.58%	63.15%	64.77%
**Kumpel^{PC}**	46.98%	43.26%	63.83%	64.58%	-	62.70%	67.69
**Kumpel^{PD}**	44.41%	43.43%	62.59%	63.15%	62.70%	-	65.09%
**Kumpel^{PE}**	46.99%	43.57%	64.01%	64.77%	67.69%	64.81%	-

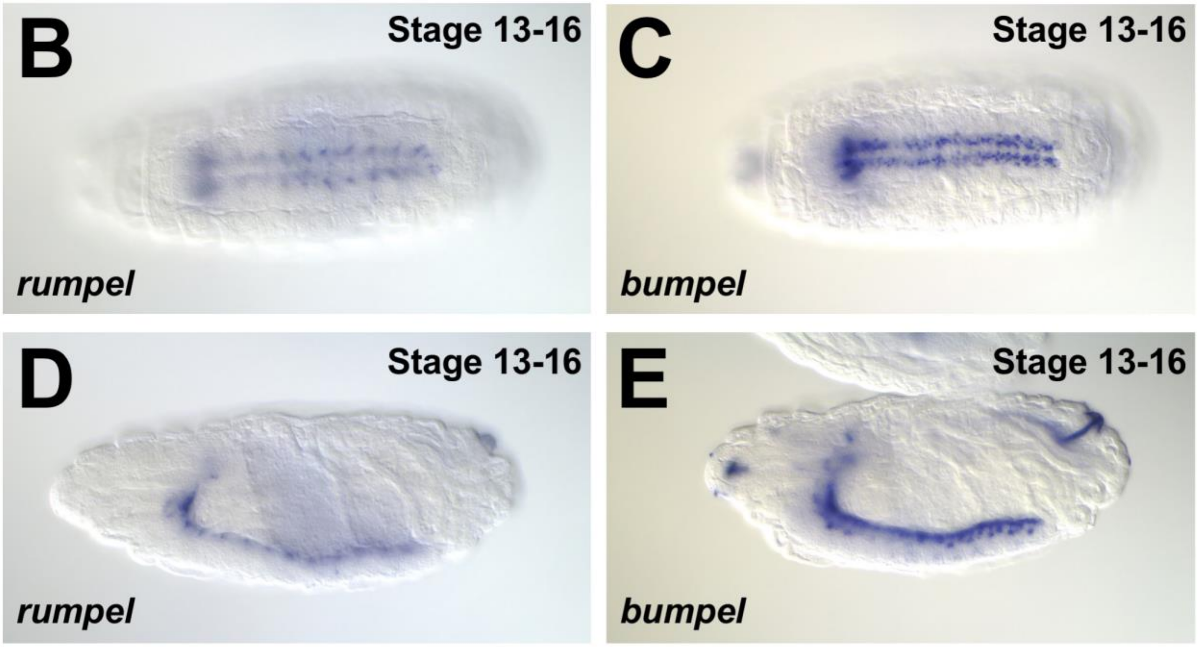

Fig. S4. Amino acid sequence comparison of Rumpel, Bumpel and Kumpel

(A) Pairwise amino acid sequence comparison between Rumpel, Bumpel and Kumpel using BLAST (ALTSCHUL et al., 1990). The homology of the different protein forms is indicated and ranges from 43% to 85% identity. The five different Kumpel isoforms are more similar on

amino acid level to each other (62-85%) than compared to Rumpel and Bumpel. (B-E) RNA *in situ* hybridizations of Drosophila embryos showing expression of *rumpel* (B,D) and *bumpel* (C,E) expression at embryonic stage 16 (images from BDGP). Both genes are expressed by the longitudinal glial cells, comprising ensheathing glia and astrocyte-like glia.

Biology Open • Supplementary information

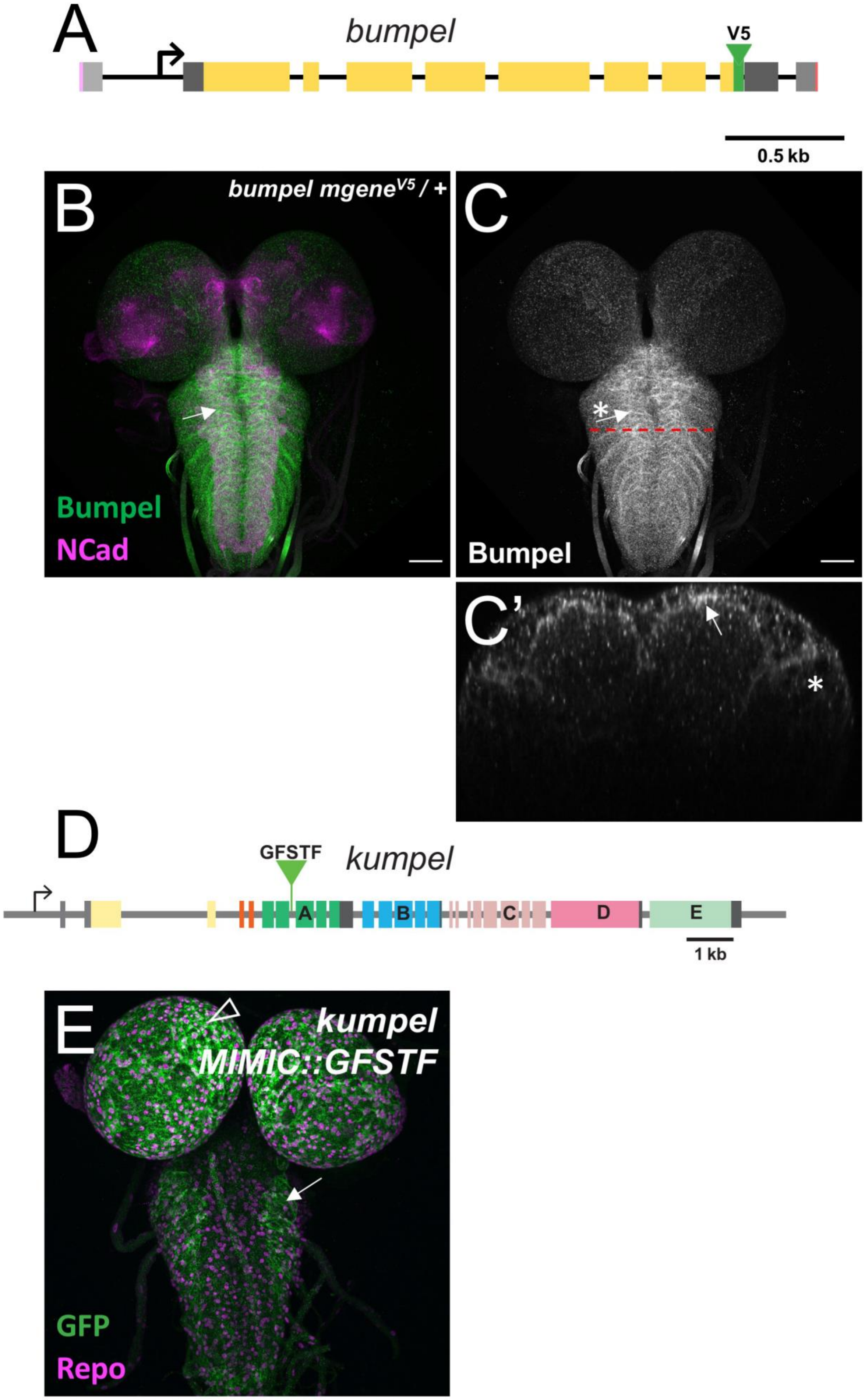

Biology Open • Supplementary information

Fig. S5. *bumpel*^{mgeneV5} and *kumpel*$^{MiMIC::GFSTF}$ show expression in CNS glial cells.

All specimens are stained for Bumpel (V5; green/grey), Kumpel (GFP; green), Repo protein localization to define glial nuclei (magenta).

(A) Schematic representation of the *bumpel* minigene carrying 500 bp upstream sequence. A V5-tag was inserted at the C-terminus just before the stop-codon. (B-C) Following anti-V5 staining, Bumpel::V5 localization (green, grey) is predominantly detected in the ensheathing (arrow) and cortex glial cells (asterisk). Anti-N-Cadherin staining is used to visualize the neuropil (magenta). The red dotted line indicates the position of orthogonal plane shown in (C'). (D) Schematic representation of the MiMIC^{GFSTF} insertion into the *kumpel* gene. (E) Kumpel protein can be detected in cortex of the brain lobes (arrowhead) and the ventral nerve cord (arrow) of the larval CNS.
